# ISR inhibition reverses pancreatic β-cell failure in Wolfram syndrome models

**DOI:** 10.1038/s41418-024-01258-w

**Published:** 2024-02-06

**Authors:** Rui Hu, Xiangyi Chen, Qiang Su, Zhaoyue Wang, Xushu Wang, Mengting Gong, Minglu Xu, Rongrong Le, Yawei Gao, Peng Dai, Zhen-Ning Zhang, Li Shao, Weida Li

**Affiliations:** 1 Medical Innovation Center and State Key Laboratory of Cardiology, Translational Medical Center for Stem Cell Therapy and Institute for Regenerative Medicine, Shanghai East Hospital, Frontier Science Center for Stem Cell Research, School of Life Sciences and Technology, Tongji University, Shanghai, 200092 China; 2 Clinical and Translational Research Center of Shanghai First Maternity & Infant Hospital, Frontier Science Center for Stem Cells, School of Life Sciences and Technology, Tongji University, Shanghai, 200092 China; 3 Department of VIP Clinic, Shanghai East Hospital, Tongji University School of Medicine, No. 1800 Yuntai Road, Pudong District, Shanghai, 200123 China; 4 Reg-Verse Therapeutics (Shanghai) Co. Ltd., Shanghai, 200120 China

**Keywords:** Experimental models of disease, Development

## Abstract

Pancreatic β-cell failure by *WFS1* deficiency is manifested in individuals with wolfram syndrome (WS). The lack of a suitable human model in WS has impeded progress in the development of new treatments. Here, human pluripotent stem cell derived pancreatic islets (SC-islets) harboring *WFS1* deficiency and mouse model of β cell specific *Wfs1* knockout were applied to model β-cell failure in WS. We charted a high-resolution roadmap with single-cell RNA-seq (scRNA-seq) to investigate pathogenesis for WS β-cell failure, revealing two distinct cellular fates along pseudotime trajectory: maturation and stress branches. *WFS1* deficiency disrupted β-cell fate trajectory toward maturation and directed it towards stress trajectory, ultimately leading to β-cell failure. Notably, further investigation of the stress trajectory identified activated integrated stress response (ISR) as a crucial mechanism underlying WS β-cell failure, characterized by aberrant eIF2 signaling in *WFS1*-deficient SC-islets, along with elevated expression of genes in regulating stress granule formation. Significantly, we demonstrated that ISRIB, an ISR inhibitor, efficiently reversed β-cell failure in *WFS1*-deficient SC-islets. We further validated therapeutic efficacy in vivo with β-cell specific *Wfs1* knockout mice. Altogether, our study provides novel insights into WS pathogenesis and offers a strategy targeting ISR to treat WS diabetes.

## Introduction

Wolfram syndrome (WS) is a rare autosomal recessive genetic disorder characterized by diabetes insipidus, diabetes mellitus, optic atrophy and deafness (also referred to as DIDMOAD) [1–4]. One of the hallmark features of WS is juvenile-onset diabetes, which presents at an average age of 6 years (range 3 weeks–16 years) [5]. The majority of WS patients require insulin treatment, resulted from selectively destroyed and functional failure of pancreatic β cells. However, due to the lack of understanding of the pathogenesis, there is currently no treatment available to reverse the progression of this disease other than provision of exogenous insulin.

The Wolframin gene (*WFS1*) encodes a protein with nine transmembrane domains across the endoplasmic reticulum (ER) membrane, which is highly expressed in pancreatic β cells and the brain [6–8]. WS is largely caused by pathogenic variants of *WFS1* [9]. Analyses of pancreas from WS patients have demonstrated a selective loss of pancreatic β cells [10]. The ER is a central cell organelle responsible for protein folding and processing. It has been reported that WFS1 located in the ER membrane plays a role in protein trafficking from the ER to the Golgi, which directly interacts with a series of vesicular cargo proteins including proinsulin [11]. *Wfs1*-deficient mice exhibit reduced insulin processing and impaired insulin secretion in response to glucose stimulation [12, 13]. Loss-of-function of *WFS1* in pancreatic β cells causes increased ER stress and activation of unfolded protein response (UPR), following pancreatic β-cell death [12, 14–16]. These findings indicate increased cell stress in *WFS1*-deficient pancreatic β cells.

Aberrant stress granule formation plays a pivotal role in various diseases, including diabetes, neurodegeneration and metabolic disorders. Stress granule formation is elicited by integrated stress response (ISR). Phosphorylation of eukaryotic initiation factor 2 (eIF2) on α subunit initiates ISR by four different kinases including protein kinase RNA-like endoplasmic reticulum kinase (PERK), which induces the activation of UPR under stress. Once ISR is triggered, the formation of the ternary complex (eIF2: GTP: methionyl-initiator-tRNA) is prevented, consequently limiting the AUG-initiated mRNA translation and inducing the assembly of stress granules and cell death [17–20]. *WFS1* loss-of-function is known to up-regulate the PERK-signaling and trigger the apoptotic pathway in pancreatic β cells [8, 14, 21–23], leading us to hypothesize that *WFS1* might control ISR and consequent stress granule formation.

Understanding of WS pathogenesis has been limited by the lack of proper human models. By modeling WS using human embryonic stem cell (hESCs) derived cerebral organoids and nerve cells, our recent study reveals Riluzole as a therapeutic molecule for WS neuropathy, demonstrating that human pluripotent stem cell derived tissues and cells could be applied as human models for WS pathogenesis investigation and drug discovery [21].

Aiming to elucidate the mechanism of pancreatic β-cell failure with *WFS1* deficiency, with similar strategy, we characterize *WFS1* deficiency through hESCs derived SC-islets as human WS β-cell failure model. Our results reveal that *WFS1* deficiency drives SC-islets β cell fate to stressed trajectory, resulting in functional failure. Moreover, we identify the ISR as the key mechanism leading to the failure of SC-islets with *WFS1* deficiency. Importantly, treatment with the ISR inhibitor, ISRIB, increases the proportion of SC-islets and mitigates apoptosis. Furthermore, ISRIB rescues impaired glucose homeostasis and increases insulin content in *Wfs1* conditional knockout mice. Thus, our study provides mechanistic insights into pancreatic β-cell disorders of WS diabetes, and proposes a potential therapeutic approach with ISRIB.

## Results

### ScRNA-seq of SC-islets reveals subpopulations including main pancreatic endocrine cells

**Fig. 1:**
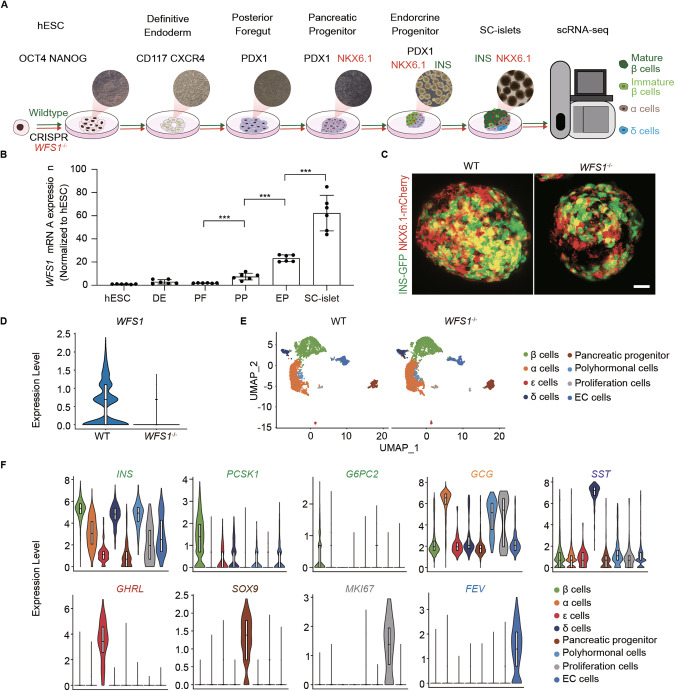
scRNA-seq analysis of SC-islets differentiated in vitro from WT and *WFS1* knockout hESCs. **A** Schematic of pancreatic islets differentiation protocol from hESCs. The key markers of the main stages are described. The red and green colors represent expression of NKX6.1-mCherry and INS-GFP, respectively. **B** Expression profile of *WFS1* (normalized to that of hESCs) during the differentiation process. *n* = 6 biological replicates for each stage. **C** Representative fluorescent images of INS-GFP and NKX6.1-mCherry in WT and *WFS1*^*−/−*^ SC-islet. Scale bars, 25 μm. **D** Violin plot showing the expression pattern of *WFS1* in WT and *WFS1*^*−/−*^ SC-islets. **E** UMAP plot of 2654 and 3397 cells from WT and *WFS1*^*−/−*^ SC-islets, respectively. Cells are colored according to their assigned type. **F** Violin plots showing the expression pattern of key marker genes of β cells, α cells, δ cells, ɛ cells, EC cells, pancreatic progenitor cells, proliferation cells and polyhormonal endocrine cells. Data are presented as mean ± SD. *p* values calculated by unpaired two-tailed Student’s *t* test were *p < 0.05, **p < 0.01, and ***p < 0.001.

*WFS1* deficiency causes childhood-onset insulin-dependent diabetes that is one of key features of WS [3, 5, 6, 24–27]. The impairment of pancreatic β-cell function and subsequent β-cell death are manifested as a result of *WFS1* loss-of-function [8, 12, 13, 28, 29]. Previous studies illustrated the role of *WFS1* associated with ER stress and UPR [8, 15, 16, 30], and *WFS1* preserves β cell function by promoting insulin synthesis and mitigating ER stress [29]. However, deeper mechanism of pancreatic β-cell failure with *WFS1* deficiency in WS diabetes remains unclear. To this end, we utilized SC-islets harboring *WFS1* deficiency as a disease model from differentiation of hESCs by stepwise differentiation in vitro [31–34]. To recapitulate WS β-cell failure from a developmental perspective, pancreatic differentiation was initiated from hESCs reporter cell line (MEL1 Nkx6.1:linker2a:mCherry; INS^*GFP/w*^) that enable to precisely and dynamically trace SC-islet β cells during various stages of differentiation [35] (Fig. 1A). Meanwhile, *WFS1* knockout hESCs reporter cell line (*WFS1*^*−/−*^) was established by using a CRISPR/Cas9 knockout strategy [31, 36]. *WFS1* knockout caused a deletion of 49 bp and consequent early stop codon by transcript frameshift (deletion from cDNA 19–67 bp, p.Pro7 Arg fs Leu126*), and did not affect self-renewal and differentiation of pluripotency (Supplementary Fig. 1) [21]. Next, we performed qRT-PCR to examine *WFS1* expression level from ES stage to SC-islet stage. We found that *WFS1* was gradually expressed during the differentiation progress in wide type (WT) cell line, consistent with its high expression in human primary pancreatic β cells as reported (Fig. 1B) [7, 8, 11, 37]. To investigate the effects of *WFS1* deficiency in SC-islets, we performed single-cell RNA sequencing (scRNA-Seq) for WT and *WFS1*^*−/−*^ SC-islets (Fig. 1C). In total, we harvested single cells of SC-islets differentiated from WT and *WFS1*^*−/−*^ hESCs for library construction, respectively. After quality control, 2654 and 3397 high-quality single-cell profiles from WT and *WFS1*^*−/−*^ SC-islets were retained for downstream analysis (Supplementary Fig. 2). We found that the expression of *WFS1* was largely diminished in *WFS1*^*−/−*^ SC-islet, confirming the complete *WFS1* knockout (Fig. 1D). Furthermore, dimensional reduction analysis using uniform manifold approximation and projection (UMAP) revealed a diversity of cell types. Unsupervised clustering analysis identified 8 distinct cell clusters in WT and *WFS1*^*−/−*^ based on the top up-regulated genes in each cluster matched to cell types in terms of published scRNA-seq analysis (Fig. 1E and Supplementary Fig. 3A) [38–41]. Clusters consisted of mainly four types of endocrine cells including β cells (especially expressing *INS*, *PCSK1* and *G6PC2*), α cells (especially expressing *GCG*), δ cells (especially expressing *SST*), ε cells (especially expressing *GHRL*), pancreatic progenitor cells (especially expressing *SOX9*), proliferation cells (especially expressing *MKI67*), EC cells (enterochromaffin cells, especially expressing *FEV*), and polyhormonal endocrine cells (co-expressing *GCG* and *INS*) (Fig. 1F, Supplementary Fig. 3B, C). Taken together, these results suggested that the utilization of SC-islet holds the promise to investigate the effect of *WFS1* in WS diabetes in vitro.

### Trajectory reconstruction reveals stressed and mature subpopulations of β cells

**Fig. 2:**
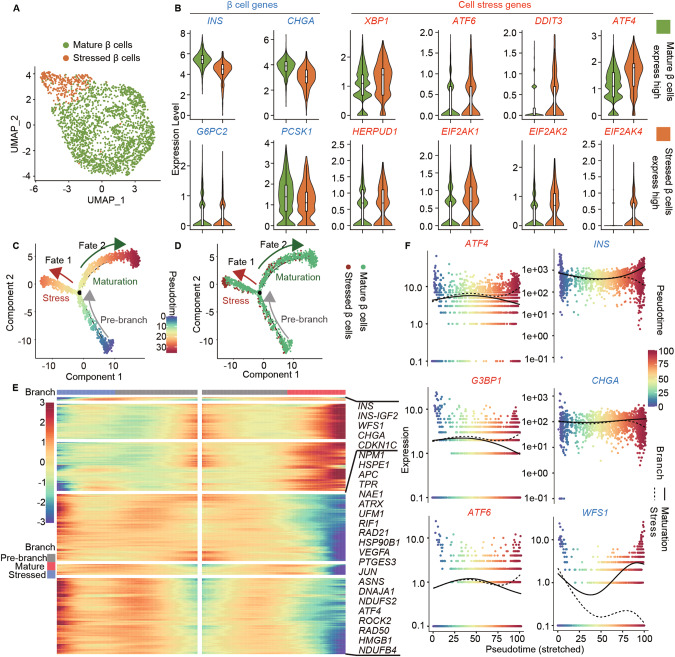
Trajectory analysis uncovers stress and maturation branches of β cells fate. **A** UMAP plot of scRNA-seq data of WT and *WFS1*^*−/−*^ SC-islet β cells. SC-islet β cells are colored and annotated into two subtypes including mature β cells and stressed β cells. **B** Violin plots showing the expression patterns of β cell marker genes (labeled in blue) and cell stress marker genes (labeled in red) between mature β cells and stressed β cells. **C**, **D** Pseudotime analysis revealing the progression of two sub-clusters of SC-islet β cells as in (**A**). Trajectory reconstruction of all single cells revealing three branches: Pre-branch, Fate 1 (indicated as stress branch) and Fate 2 (indicated as maturation branch), defined by expression profiles. Cells are colored by pseudotime (**C**) and SC-islet β cell subtypes (**D**). **E** Heatmap showing the expression of the top 500 differently expressed genes in three branches as in (**C)**, highlighting representative genes associated with pancreatic β cells specific genes and cell stress genes along the right margin. From the center to the left of the heatmap, the curve from the pre-branch along the trajectory to fate 1 branch. From the center to the right, the curve from pre-branch to fate 2 branch. **F** The expression dynamics of selected genes from pre-branch to stress and maturation branches, respectively. Each point represents one cell. Cells are colored by pseudotime.

Pancreatic β cells are essential in maintaining glucose homeostasis by regulating insulin secretion in response to glucose stimulation [42]. Besides regulating cellular function, extensive researches have revealed significant heterogeneity within the pancreatic β-cell population [43–45]. To investigate the function of *WFS1* in SC-islets β cell heterogeneity, we further re-clustered all merged SC-islet β cells using dimensionality reduction and unsupervised clustering into two main β cell subpopulations (Fig. 2A). We identified these two subpopulations according to top up-regulated genes and defined into mature β cells and stressed β cells. Mature β cells were characterized by high expression of mature pancreatic β cell markers such as *INS*, *CHGA*, *G6PC2*, and *PCSK1*, whereas stressed β cells showed high expression of stress-associated markers [19, 46] such as *XBP1*, *ATF4*, *ATF6*, *DDIT3*, *HERPUD1*, *EIF2AK1*, *EIF2AK2*, and *EIF2AK4* (Fig. 2B). Together, we unveiled the heterogeneity with two subpopulations including mature β cells and stressed β cells.

Furthermore, to decipher the trajectory of SC-islets β cell fate, single-cell pseudotemporal analysis was performed to model the cell fates in SC-islet β cells. By utilizing Monocle [47–49] pseudotime analysis to determine the transcriptional fate, we found two different branches in SC-islet β cells (Fig. 2C). We attempted to elucidate the molecular dynamics to distinguish the two branches. The analysis of gene expression dynamics was focused on the top 500 differentially expressed genes (Fig. 2E and Supplementary Table 3). We found that stress-associated markers were highly expressed along fate 1 branch, such as *ATF4* and *JUN* [50, 51], while SC-islets β cell identity and maturation markers were highly expressed along fate 2 branch, such as *INS*, *INS-IGF2*, *CHGA* and *CDKN1C* [52]. On the basis of the above features, we inferred fate 1 branch as stress state, and fate 2 branch as maturation state (Fig. 2C). Notably, total SC-islet β cells branched opposite divergent ends as two terminally differentiated cell types. Most of stressed β cells were found at the terminal end of fate 1 branch (stress branch), consistent with stressed β cell population in SC-islets (Fig. 2D). Meanwhile, we assessed the gene expression patterns associated with pancreatic β cell markers and stress-associated markers during two fate branches. The expression of pancreatic β cell markers, including *INS* and *CHGA* increased along the maturation branch. Notably, *WFS1* was highly expressed along the maturation branch, indicating the function of *WFS1* correlated to β-cell maturation. Whereas, the expression of *ATF4*, *G3BP1* and *ATF6* increased along the stress branch but notably decreased along the maturation branch (Fig. 2F). Taken together, through single-cell transcriptomic analysis, the composition of SC-islet β cells was identified into two different subpopulations including mature and stressed β cells, characterized with two distinct fate trajectories.

### *WFS1* deficiency directs β cells into the stress branch lack of functional maturation

**Fig. 3:**
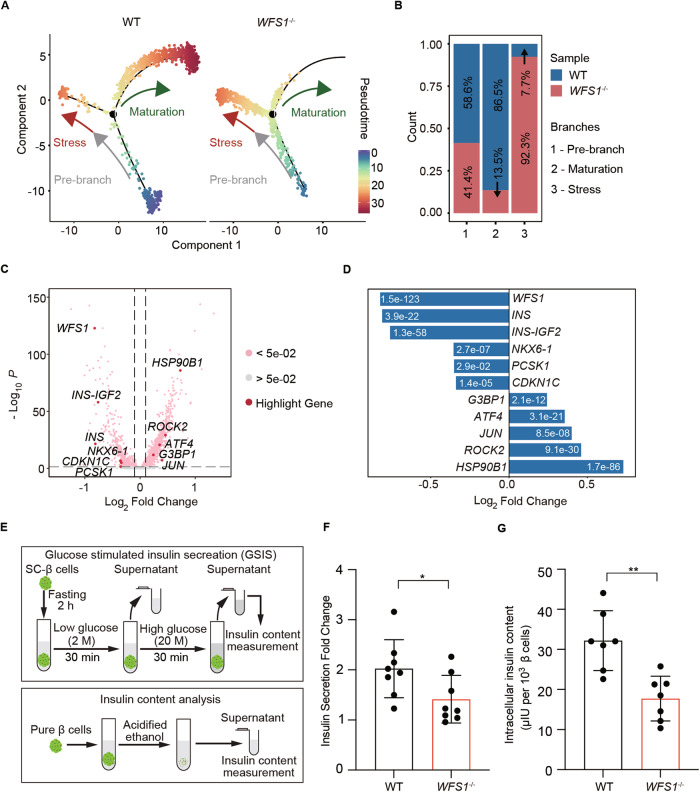
WFS1 deficiency impairs β cell maturation and function. **A** Pseudotime analysis showing the progression of SC-islet β cells in WT (left panel) and *WFS1*^*−/−*^ SC-islet β cells (right panel), respectively. Cells are colored by pseudotime. **B** Bar plots presenting the percentage of stressed β cells or mature β cells in three trajectory branches. Blue represents WT and red represents *WFS1*^*−/−*^. **C** Volcano plot of differential expression analysis between WT and *WFS1*^*−/−*^ SC-islet β cells. Each dot represents a gene, and selected DEGs are highlighted in red. **D** Particular up-regulated and down-regulated genes by log-fold change in *WFS1*^*−/−*^ SC-islet β cells with adjusted *p* < 0.05. **E** Schematic of in vitro function assays and insulin content test in WT and *WFS1*^*−/−*^ SC-islets. **F** GSIS in vitro of WT and *WFS1*^*−/−*^ SC-islets, *n* = 8 per group. **G** Insulin content of WT and *WFS1*^*−/−*^ SC-islets, *n* = 7. Data are presented as the mean ± SD. *p* values calculated by unpaired two-tailed Student’s *t* test for (**F**) and (**G**) were *p < 0.05 and **p < 0.01.

To investigate the effect of *WFS1* on cell fate, we performed Monocle pseudotime analysis for WT and *WFS1*^*−/−*^ SC-islet β cells, respectively. Combining with defined two cell fate branches, we found that the pseudotime trajectory began with pre-branch and then mostly placed at maturation branch among WT SC-islet β cells. Of note, *WFS1*^*−/−*^ SC-islet β cells mainly placed at the stress branch along the pseudotime trajectory, which were stagnated towards the maturation branch (Fig. 3A). Next, we assessed the proportions of WT and *WFS1*^*−/−*^ SC-islet β cells in three cell fate branches (Fig. 3B). The percentage of WT and *WFS1*^*−/−*^ SC-islet β cells exhibited about 58.6% and 41.4% of total cells in pre-branch, respectively. The proportion of WT cells reached 86.5% at the maturation branch, whereas the proportion of *WFS1*^*−/−*^ cells accounted for 92.3% of total cells in the stress branch (Fig. 3B), which was consistent with the stagnant trajectory of *WFS1*^*−/−*^ SC-islet β cells towards the maturation branch. Meanwhile, we performed a deeper analysis of transcriptomic changes in WT and *WFS1*^*−/−*^ SC-islet β cells to detect the molecular changes. Differential expression analysis generated a large number of differential expression genes (DEGs) that comprised a distinctive gene expression profile with 2701 up-regulated and 446 down-regulated genes in *WFS1*^*−/−*^ SC-islet β cells (Fig. 3C and Supplementary Table 4). We found that the expression of genes related to pancreatic β cell maturation and function such as *INS*, *NKX6.1*, *PCSK1*, and *CDKN1C* were down-regulated, and cell stress-associated genes such as *ATF4*, *JUN*, *ROCK2* and *HSP90B1* were highly up-regulated in *WFS1*^*−/−*^ SC-islet β cells (Fig. 3C). A summary of the significant up-regulated or down-regulated DEGs within *WFS1*^*−/−*^ SC-islet β cells was shown (Fig. 3D). Mature pancreatic β cells gain the capacity of glucose-stimulated insulin secretion (GSIS) [53].By contrast, our analysis of scRNA-seq data showed that *WFS1*^−/−^ SC-islet β cells exhibited stressed cell fate lack of maturation. To validate this, we performed GSIS assays in WT and *WFS1*^*−/−*^ SC-islets. Insulin secretion upon glucose stimulation was measured by scoring the ratio of insulin release in high glucose to that in low glucose [31]. Compared with WT SC-islets, *WFS1*^*−/−*^ SC-islets showed significantly reduced insulin secretion (Fig. 3E, F). And the insulin content per cell was measured in *WFS1*^*−/−*^ SC-islets and result revealed significantly lower intracellular insulin content compared with WT SC-islets (Fig. 3E, G). Collectively, our result revealed that *WFS1* was required for advancing cell fate of β cells towards the maturation trajectory.

### *WFS1* deficiency induces ISR in β cells

**Fig. 4:**
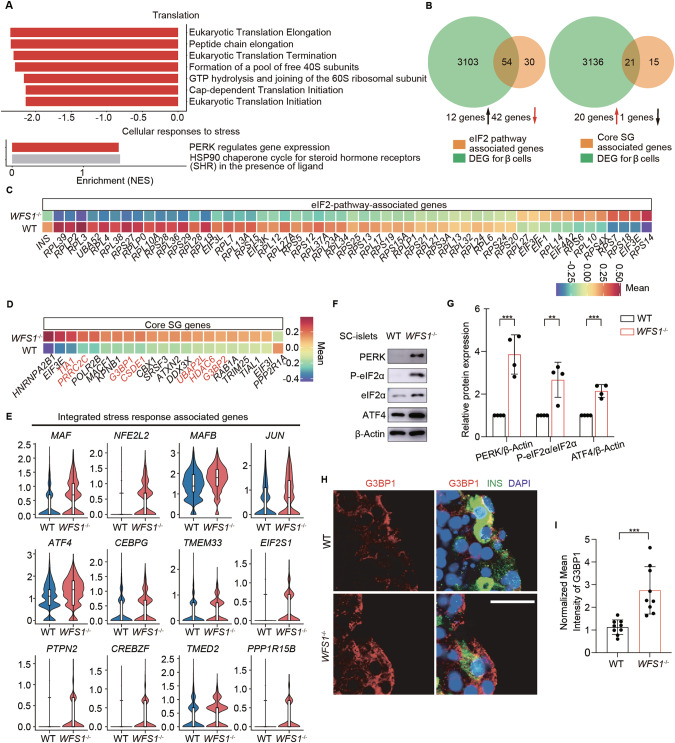
Single-cell transcriptional analysis reveals activated ISR in *WFS1*-deficient β cells. **A** Reactome pathway enrichment analysis of differentially expressed genes from *WFS1*^*−/−*^ SC-islet β cells using GSEA. Enrichment analysis showed that protein translation processes were enriched in down-regulated genes, whereas the stress pathways were enriched in up-regulated genes. **B** Venn diagrams showing the overlapping genes between *WFS1*^*−/−*^ SC-islet β cell DEGs and eIF2-pathway-associated genes, between *WFS1*^*−/−*^ SC-islet β cell DEGs and stress granule-associated genes, respectively. Up-regulated and down-regulated genes in overlapping genes were highlighted. **C** Average expression of signature genes in eIF2-pathway-associated genes overlapped with DEGs as in (**B)**. **D** Average expression of signature genes in stress granule-associated genes overlapped with DEGs as in (**B)**. Genes described as “essential” to SG assembly were labeled in red. **E** Violin plots showing the expression patterns of the ISR associated genes in WT and *WFS1*^*−/−*^ SC-islet β cells. **F**, **G** PERK, Phosphorylation of eIF2a and ATF4 pattern were determined by western blot analysis in WT and *WFS1*^*−/−*^ SC-islets. **H**, **I** Immunostaining of G3BP1 in WT and *WFS1*^*−/−*^ SC-islets. Scale bars, 25 μm (**H**) and mean intensity measurements for G3BP1, *n* = 9 (**I**). Data are presented as the mean ± SD. *p* values calculated by unpaired two-tailed Student’s *t* test were *p < 0.05, **p < 0.01, and ***p < 0.001.

To determine the signaling pathways regulating *WFS1*^*−/−*^ SC-islets β cell functional failure, we performed gene set enrichment analysis (GSEA) on the SC-islet β cells and the top enriched pathways were identified (Supplementary Table 5). We found that Reactome pathways related to translation such as eukaryotic translation elongation, peptide chain elongation, eukaryotic translation termination, formation of a pool of free 40 S subunits, GTP hydrolysis and joining of the 60S ribosomal subunit, cap-dependent translation initiation and eukaryotic translation initiation were significantly enriched in *WFS1*^*−/−*^ SC-islet β cells among top 25 down-regulated pathways collected from the pathway enrichment analysis. Meanwhile, Reactome pathways related to cellular response to stress such as PERK regulates gene expression and HSP90 chaperone cycle for steroid hormone receptors in the presence of ligand were significantly enriched in *WFS1*^*−/−*^ SC-islet β cells among top 25 up-regulated pathways in the pathway enrichment analysis (Fig. 4A, Supplementary Fig. 4). To further verify the down-regulated translation in *WFS1*^*−/−*^ SC-islet β cells, we compared genes expression involved in the eIF2 signaling pathway [50, 54, 55] between WT and *WFS1*^*−/−*^ SC-islet β cells. 54 shared genes associated with eIF2 signaling pathway among DEGs within *WFS1*^*−/−*^ SC-islet β cells were significantly enriched (Fig. 4B). The comparative heatmap of relative expression changes in eIF2 signaling pathway associated genes showed that 75% (42 genes in 54 shared genes) genes were down-regulated, indicating activation of the ISR (Fig. 4C). Stress granules are formed when translation initiation is inhibited by stress responses [56, 57]. Furthermore, we evaluated the core stress granule genes [58] between WT and *WFS1*^*−/−*^ SC-islet β cells. 21 shared genes associated with core stress granule genes among DEGs within *WFS1*^*−/−*^ SC-islet β cells were significantly enriched (Fig. 4B). Notably, 20 genes in total shared 21 genes associated with core stress granule genes were significantly up-regulated, including all seven genes that have been described as “essential” to stress granule assembly [58] (Fig. 4D). Previous study had shown that PERK phosphorylates eIF2α to activate the ISR, leading to a decrease in global protein translation [19, 59]. Consistently, we found that the expression of PERK pathway regulated genes was significantly up-regulated and the process of intracellular translation was significantly down-regulated in *WFS1*^*−/−*^ SC-islet β cells. Meanwhile, we also examined the gene expression levels associated with the UPR, and found the elevated expression pattern of the UPR associated genes (Supplementary Fig. 5). Taken together, our results revealed that *WFS1* deficiency activates the ISR in SC-islet β cells with global attenuation of translation. To further verify the activation of ISR in *WFS1*^*−/−*^ SC-islet β cells, we tested the expression of ISR associated genes. We found that the expression of ISR associated genes was highly increased in *WFS1*^*−/−*^ SC-islet β cells compared with WT (Fig. 4E). The expression levels of key factors of PERK/eIF2 signaling were examined by western blot, revealing significantly increased phosphorylation of eIF2α, and enhanced protein levels of PERK and ATF4 in *WFS1*^*−/−*^ SC-islets compared with WT (Fig. 4F, G, Supplementary Fig. 6). Meanwhile, we applied immunostaining to examine the expression of G3BP1, which is an essential component of stress granules. The mean intensity of stress granules in INS^+^ cells was significantly higher in *WFS1*^−/−^ SC-islets compared with WT (Fig. 4H, I).

### ISR inhibitor reverses pancreatic β-cell failure

As shown above, ISR is activated by *WFS1* deficiency in β cells. To investigate whether inhibition of ISR could be applied to treat pancreatic β-cell failure in WS, we tested the ability of ISRIB (an ISR inhibitor [60, 61]) both in *WFS1*-deficient SC-islets and pancreatic *Wfs1*-deficient mice.

**Fig. 5:**
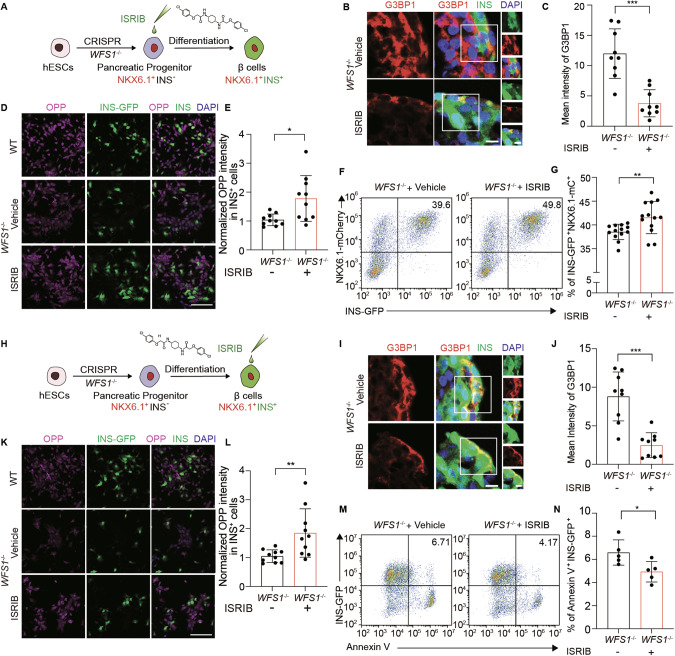
ISR inhibitor ISRIB increases β cell proportion treated from pancreatic progenitor cell stage and reduces β cell apoptosis from SC-islets stage. **A** Schematic diagram of ISRIB treatment starting from pancreatic progenitor stage. Immunostaining of G3BP1 in *WFS1*^*−/−*^ SC-islets treated with ISRIB or vehicle. Scale bars, 10 μm (**B**) and mean intensity measurements for G3BP1, *n* = 9 (**C**). OPP protein synthesis assay of WT and *WFS1*^*−/−*^ SC-islets treated with ISRIB or vehicle, Scale bars, 100 μm (**D**) and mean intensity measurements for OPP in INS^+^ β cells, *n* = 10 (**E**). Representative FACS plots (**F**) and quantifications (**G**) of INS-GFP^+^/NKX6.1-mCherry^+^ populations in *WFS1*^*−/−*^ SC-islets treated with ISRIB or vehicle starting from pancreatic progenitor stage, n = 13. **H** Schematic diagram of ISRIB treatment in SC-islets. Immunostaining of G3BP1 in *WFS1*^*−/−*^ SC-islets treated with ISRIB or vehicle. Scale bars, 10 μm (**I**) and mean intensity measurements for G3BP1, *n* = 9 (**J**). OPP protein synthesis assay of WT and *WFS1*^*−/−*^ SC-islets treated with ISRIB or vehicle, Scale bars, 100 μm (**K**) and mean intensity measurements for OPP in INS^+^ β cells, *n* = 10 (**L**). Representative FACS plots (**M**) and quantifications (**N**) of Annexin V^+^/INS-GFP^+^ populations in WT and *WFS1*^*−/−*^ SC-islets treated with ISRIB or vehicle, *n* = 5. Data are presented as the mean ± SD. *p* values calculated by unpaired two-tailed Student’s *t* test were *p < 0.05, **p < 0.01 and ***p < 0.001.

First, from PP cell stage to SC-islets stage, *WFS1*-deficient SC-islets were treated with ISRIB at the concentration of 100 nM (Fig. 5A). The mean intensity of G3BP1 was significantly decreased in *WFS1*-deficient SC-islets treated with ISRIB as compared to vehicle (Fig. 5B, C). Since activated ISR inhibits protein synthesis, we performed nascent polypeptide synthesis assay *via* O-propargyl-puromycin (OPP) labeling to assess total protein synthesis [62–65]. As a result, the decreased total protein synthesis was significantly restored in ISRIB-treated *WFS1*-deficient SC-islets as compared to control (Fig. 5D, E). Furthermore, we found that the percentage of INS-GFP NKX6.1-mCherry double positive SC-islet β cells in *WFS1*^*−/−*^ SC-islet treated with ISRIB was significantly increased as compared to vehicle (Fig. 5F, G). Next, we treated *WFS1*-deficient SC-islet with 100 nM ISRIB or vehicle from SC-islets stage for 2 days (Fig. 5H). As compared to vehicle, the mean intensity of G3BP1 in *WFS1*-deficient SC-islets treated with ISRIB was significantly decreased, indicating the reduced formation of stress granules (Fig. 5I, J). Consistently, total protein synthesis was also restored with ISRIB treatment (Fig. 5K, L). Meanwhile, the apoptosis of SC-islet β cells was significantly reduced in *WFS1*-deficient SC-islet treated with ISRIB as compared to vehicle (Fig. 5M, N). These results suggested that ISR inhibition reverses pancreatic β-cell loss.

### ISR inhibitor improves glucose homeostasis in *Wfs1* pancreatic conditional knockout mice

**Fig. 6:**
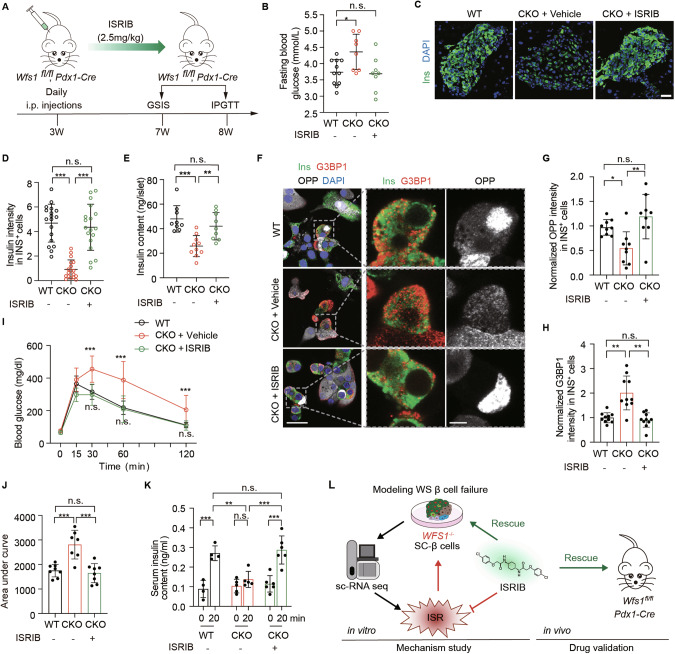
ISR inhibitor ISRIB reverses pancreatic β cells functional failure in *Wfs1* conditional knockout mice. **A** Schematic of the strategy of generating pancreatic *Wfs1* conditional knockout mice for ISRIB treatment. **B** Fasting glucose levels in WT and CKO mice after ISRIB treatment, *n* ≥ 8. Immunostaining of Ins (**C**) and quantifications (**D**) of Ins intensity in WT and CKO mice after ISRIB treatment, *n* = 18 islets from *n* = 3 mice. Scale bar, 25 μm. **E** Insulin content in WT and CKO mice after ISRIB treatment, *n* = 9. OPP protein synthesis assay of WT and CKO mice after ISRIB treatment, Scale bars, 25 μm (**F,** left) and 5 μm (**F,** right**)** and mean intensity measurements for OPP (**G**) and G3BP1 (**H**) in β cells, *n* = 9 from *n* = 4 mice (**G**, **H**). IPGTT (**I**) and AUC analysis (**J**) in WT and CKO mice after ISRIB treatment, *n* ≥ 7. **K** GSIS in WT and CKO mice after ISRIB treatment, *n* ≥ 4. **L** Schematic diagram showing activated ISR in *WFS1*-deficient β cells and reversed β cell function treated with ISRIB in vitro and in vivo. Data are presented as the mean ± SD. *p* values calculated by unpaired two-tailed Student’s *t* test for (**B)**, (**D)**, **(E)**, (**G),** (**H),** and (**I)**; two-way ANOVA with Sidak’s test for multiple comparisons for (**H)** and (**J)**. n.s. not significant, *p < 0.05, **p < 0.01 and ***p < 0.001.

To test the in vivo efficacy of ISRIB, we generated pancreatic *Wfs1* conditional knockout mice by crossing the *Wfs1-flox* mice with *Pdx1-Cre* mice (*Wfs1*^*fl/fl*^, *Pdx1-Cre* mice; CKO mice) (Supplementary Fig. 7A) [21, 37]. We administered 2.5 mg/kg ISRIB or vehicle in CKO mice through intraperitoneal (i.p.) injections from 3 to 8 weeks old (Fig. 6A). We observed a significantly decreased fasting glucose level in *Wfs1*-deficient mice treated with ISRIB as compared to vehicle at 8 weeks old (Fig. 6B). To investigate the intracellular changes of pancreatic β cells, we performed the immunostaining of insulin in mouse pancreas. We found that insulin intensity and insulin content of pancreatic β cells in ISRIB-treated CKO mice were significantly restored as compared to vehicle, similar to that in WT mice (Fig. 6C–E). Consistently, OPP labeling showed that ISRIB treatment significantly restored the impaired total protein synthesis, and reduced stress granule formation in CKO mice (Fig. 6F–H).

Furthermore, intraperitoneal glucose tolerance test (IPGTT) showed that ISRIB treatment significantly improved glucose tolerance in CKO as compared to vehicle-treated mice (Fig. 6I, J). Meanwhile, we examined the insulin secretion by GSIS in vivo. We found that ISRIB-treated CKO mice showed significantly improved insulin secretion upon high glucose stimulation as compared to vehicle (Fig. 6K). Notably, there was no significant difference of insulin secretion upon high glucose stimulation between WT and ISRIB-treated CKO mice (Fig. 6K). To explore the potential toxicity of ISRIB in the body, we detected the histological images of major organs including heart, liver, spleen, lung and kidney. No inflammatory cell infiltration and histopathological changes were observed with ISRIB treatment (Supplementary Fig. 7B). These results demonstrated that ISRIB could reverse β-cell failure and improve glucose homeostasis in vivo. Overall, our results suggested that ISRIB could be applied to treat pancreatic β-cell failure and function in WS diabetes (Fig. 6L).

## Discussion

The clinical progression of WS diabetes is characterized by severe β-cell loss during juvenile stage, which ultimately becomes resistant to treatment [2, 5]. Unfortunately, the lack of understanding regarding its pathogenesis has hindered the development of effective therapies for this disease. The investigation of WS diabetes has been limited due to ethical concerns and the scarcity of human samples. The effect of *WFS1* deficiency on pancreatic β-cell function has been modeled in rodents [12, 13, 29, 66, 67]. Meanwhile, the phenotypes resulted from *WFS1* loss-of-function have also been analyzed in cell lines and SC-islets [16, 28, 68]. However, the underlying mechanisms of *WFS1* deficiency causing β-cell functional failure and loss are elusive, remaining as a challenge for WS drug discovery. To achieve a deeper understanding of WS diabetes, we applied SC-islets differentiated from *WFS1* knockout hESCs as human model. Our investigation revealed the presence of two distinct cell fate trajectories in β cells: the maturation branch and the stress branch. Notably, *WFS1* deficiency blocked β-cell fate trajectory to maturation and directed them towards stress trajectory. Further analysis identified the activated ISR as a crucial pathogenic mechanism for WS β-cell failure.

WFS1 is a protein with nine transmembrane domains across the ER membrane, which is highly expressed in pancreatic β cells and brain [6–8]. Previous studies using rodent models mainly focused on the relationship between the ER stress and pancreatic β cell function. It has been reported that loss-of-function of *WFS1* results in pancreatic β-cell failure by eliciting chronic ER stress [12, 13, 16, 28, 66, 67]. To date, significant efforts have been devoted to treat WS by decongesting ER stress [28]. In rodent and SC-islet models, ER stress mitigators, such as valproic acid and GLP-1 receptor agonists have been proposed as potential drug candidates [69–72]. Several studies have suggested *WFS1* deficiency causes calcium dyshomeostasis, thus compounds targeting calcium signaling such as dantrolene sodium (an ER Ca^2+^ stabilizer), ibudilast and calpain inhibitor were also identified as drug candidates to treat with WS [73–75]. Nevertheless, given the fact that calcium is a universal second messenger ubiquitously existing in various cell types, application of calcium stabilizers is limited due to their potential for systemic side effects [76]. Besides targeting ER stress and calcium, it is of substantial importance to identify alternative therapeutic targets and molecules to decongest stress in *WFS1*-deficient β cells. Here, by targeting the activated ISR in *WFS1*-deficient SC-islets, we found ISRIB, an ISR inhibitor [60, 61, 77–79], efficiently reduced stress granule formation and cell death in SC-islet β cells. Moreover, we validated in vivo efficacy of ISRIB by observing its effects in pancreatic *Wfs1* conditional knockout mice. ISRIB treatment led to a significant restoration of insulin content and β cell mass. *Wfs1*-deficient CKO mice treated with ISRIB exhibited improved glucose homeostasis. These results, obtained from both in vitro human model and in vivo animal model, suggested that ISRIB could be a potential drug candidate for the treatment of WS diabetes.

The mechanism of stress granules assembly mediated by phase separation is not fully understood [80]. Our results demonstrated that *WFS1* deficiency up-regulated stress granule-associated gene such as *G3BP1*, indicating that *WFS1* deficiency enhances stress granule formation. Previous studies have shown ISR leads to a decreased global protein translation and the induction of specific gene expression, including *ATF4* [19, 59]. ATF4 acts as an effector of the ISR, regulating stress-responsive gene expression [81–87]. In *WFS1*-deficient SC-islet β cells, we observed increased expression of G3BP1, which is a crucial component for stress granule. This finding suggested that ATF4, up-regulated by *WFS1* deficiency mediated ISR, might activate *G3BP1* transcriptional expression. Further investigation is needed to confirm this. Moreover, we also discovered that overexpression of *WFS1* alleviated the phosphorylation of eIF2α and stress granule assembly in β cells (data not shown). Our discoveries indicate a critical role of *WFS1* in stress granule formation and emphasize the need for future research in this area.

Besides diabetes, neuropathy is also a major manifestation for WS. As recently reported, dysregulated ISR signaling contributes to the pathogenesis of several neurodegenerative diseases [19, 88, 89], and the effect of ISRIB on the neurodegeneration has been tested in amyotrophic lateral sclerosis, brain injury and age-related memory decline [61, 78, 79, 89–91]. Combined with our result, it will be valuable for further study to test therapeutic effect of ISRIB on WS manifestations of both diabetes and neurodegeneration in clinical trial. Moreover, with similar strategy to model WS neuropathy by application hESCs derived cerebral organoids and neural cells, we demonstrate that *WFS1* deficiency delays neuronal differentiation with disrupted expression of genes associated with psychiatric disorders, impairs synapse formation, and renders astrocytes toxic to neurons by reducing EAAT2 (Glutamate transporter). On the basis of this, we demonstrate Riluzole as a therapeutic molecule to reverse neuronal loss and abnormality of synapse formation associated with WS neuropathy and further validate its efficacy in vivo in animal model [21]. Altogether, in future study, it would be intriguing to explore the synergistic curative effect of combining Riluzole with ISRIB for WS treatment.

## Materials and methods

### Generation of WFS1 knockout hESCs with CRISPR/Cas9

One or two sgRNAs inserts targeting *WFS1* gene were cloned into vector containing U6 promoter and CMV promoter drove hCas9 (vector as a gift from Prof. Zhili Rong, Southern Medical University, Guangzhou, China). MEL1 Nkx6.1:linker2a:mCherry; INS^*GFP/w*^ gene-edited cell line was provided by Dr. Xin Cheng. All cell lines were checked to be mycoplasma-free once a month by PCR assay. Mel1-Reporter line was transfected with above-mentioned plasmid carrying sgRNAs and hCas9. After 24–48 h of electroporation, puromycin was added into hESCs culture medium for selection as concentration of 400 ng/ml, and hESCs culture medium with puromycin was replaced after 2 days selection. Cell clones were picked and cultured for 5–7 days later. PCR was used to validate the *WFS1* knockout cell line.

### In vitro differentiation of SC-islets derived from hESCs

hESCs were differentiated into SC-islets as described previously [31, 33, 92]. In brief, hESCs were cultured on the Matrigel-coated plate to start differentiation. Then the differentiation process contained planar culture through stage 1 to stage 4 to generate NKX6.1^+^ progenitor cells and 3D culture from stage 5 to stage 7 to yield mature SC-islets. SC-islets differentiation that did not pass a minimum criteria of 30% INS^+^/mCherry^+^ double positive cells at the final stage were excluded from further analysis. No data were excluded for other analyses of this study, and the randomization was not applicable. The in vitro experiments, mean intensity measurement, cell type and apoptosis quantification were blinded to investigator to analyze.

### Library construction and NGS sequencing

DNBelab C Series High-throughput Single-Cell RNA Library (MGI, #940-000047-00) was utilized for scRNA-seq library preparation. In brief, the single-cell suspensions were converted to barcoded scRNA-seq libraries through steps including droplet encapsulation, emulsion breakage, mRNA captured beads collection, reverse transcription, cDNA amplification and purification. cDNA production was sheared to short fragments with 250-400 bp, and indexed sequencing libraries were constructed according to the manufacturer’s protocol. Qualification was performed using Qubit ssDNA Assay Kit (Thermo Fisher Scientific) and Agilent Bioanalyzer 2100. All libraries were further sequenced by the MGISEQ-2000RS with pair-end sequencing. The sequencing reads contained 30-bp read 1 (including the 10-bp cell barcode 1, 10-bp cell barcode 2 and 10-bp unique molecular identifiers (UMI)), 100-bp read 2 for gene sequences and 10-bp barcodes read for sample index. Generation of FASTQ files were carried out using DNBelab_C4_scRNA_v2.3.

### ScRNA-seq data processing (alignment, barcode assignment, and UMI counting)

The sequencing data were processed using an open-source pipeline (https://github.com/MGI-tech-bioinformatics/DNBelab_C_Series_scRNA-analysis-software). Briefly, all samples were performed sample de-multiplexing, barcode processing, and single-cell 3’ unique molecular identifier (UMI) counting with default parameters. Processed reads were then aligned to GRCh38 genome reference using STAR (2.5.1b). Valid cells were automatically identified based on the UMI number distribution of each cell by using the “barcodeRanks()” function of the DropletUtils tool to remove background beads and the beads that had UMI counts less than the threshold value. Finally, we used PISA to calculate the gene expression of cells and create a gene x cell matrix for each library.

### ScRNA-seq data analysis

Raw sequencing data were aligned to the GRCh38 human reference genome. We processed the resulting data using Seurat [93] and removed low-quality cells with less than 200 or more than 9000 detected genes, or mitochondria gene content was more than 10%. Genes were filtered with less than 3 cells detected in each sample. SCTransform-v2 was used for normalization. SelectIntegrationFeatures and PrepSCTIntegration functions were used to perform integration. RunPCA function in Seurat was used for principal component analysis and selected 40 PCs for cell clustering and downstream analysis. FindNeighbors and FindClusters (the resolution was adjusted to 0.8) functions were run to get 11 clusters. Dimensionality reduction was performed by RunUMAP function in Seurat. Marker genes were identified of each cluster to define cell types using FindAllMarkers in Seurat. According to known gene markers including *GCG, INS*, *SST*, *CHGA*, *GHRL*, *FEV*, we defined those different clusters. Finally, we merged 11 clusters to 8 cell types including α cells, β cells, δ cells, ε cells, polyhormonal cells, pancreatic progenitor, EC cells and proliferation cells. We re-identified marker genes for 8 cell types and highlighted top 10 positive marker genes per cell type. We compared the differential expression pattern in different cell types between WT and *WFS1*^*−/−*^. Differential expression genes (DEGs) were identified using FindMarkers function in Seurat with adjusted *p* < 0.05 and |log_2_-fold change| > 0.1 as cutoffs. β cells were further classified into 3 sub-clusters by re-running steps above. 3 sub-clusters were merged to 2 subtypes according to β cell identity markers and stress markers. DEGs between 2 β cell types were identified using Findmarkers function in Seurat. Visualizations were generated using Seurat, ggplot2 (https://ggplot2.tidyverse.org) and pheatmap (https://github.com/raivokolde/pheatmap).

### Pathway analysis

Pathway analysis was based on Reactome pathway database using R package clusterProfiler [94, 95]. All genes ranked by the fold change calculated above between WT and *WFS1*^*−/−*^ were considered.

### Pseudotime analysis

R package Monocle2 was used to order β cells and progenitor cells for pseudotime analysis. SetOrderingFilter function was used to get a list of gene ids to be used for ordering. Reducing the Dimensionality of data was performed by ReduceDimension function. DDRTree was used to learn tree-like trajectories. OrderCells function was used to ordering cells along the trajectory. The roots of these trajectories were determined by the expression of unique genes. The trajectory plots were generated by plot_cell_trajectory function. Beam function was used to figure out genes differentially expressed between the branches. Genes were selected with *q* < 1e-4 as cutoff. The heatmap were generated by plot_genes_branched_heatmap function with top 500 genes selected.

### Quantitative real-time PCR

Total RNA was extracted using the TRNzol Universal kit and reverse transcribed into cDNA using Quantscript RT Kit (TianGen). Quantitative real-time PCR was carried out using SuperReal SYBR Green kit on LightCycler 96 (Roche). The amplification efficiency for each primer and the cycle threshold were determined automatically by Lightcycler software (Roche). The fold-change was calculated by the comparative CT (2^−ΔΔCT^) method against GAPDH. Primer sequences were shown in Supplementary Table 1.

### Immunofluorescence staining and quantification of immunofluorescence image

SC-islets were rinsed once with precooled PBS and fixed with 4% paraformaldehyde overnight at 4 °C and washed with DPBS for three times. SC-islets were then embedded in optimum cutting temperature (O.C.T) for cryosectioning at 8 µm with Leica CM1950. Mouse pancreases were collected in PBS, fixed in 4% paraformaldehyde on ice for 1 h, washed in PBS for three times, and dehydrated in 30% sucrose at 4 °C overnight. Mouse pancreases were embedded in O.C.T for cryosectioning at 10 µm with Leica CM1950. Next, for SC-islets or mouse pancreases, sections were permeabilized with PBST (PBS with 0.1% Triton X-100) for 15 min and then blocked with PBS containing 5% donkey serum for 2 h at RT. Primary antibodies were added at appropriate dilutions at 4 °C overnight. The next day, cells were washed with PBS for three times, secondary antibodies were added at appropriate dilutions for 1 h at RT. Cell nuclei were stained with DAPI. Primary antibodies used in this study included: WFS1 (Abcam, ab259362, 1:50), INSULIN (Sigma, I2018, 1:500), G3BP1 (Proteintech, 13057-2-AP, 1:500). Secondary antibodies were available from Jackson ImmunoResearch Laboratories. Images were captured with Leica SP8 confocal microscope. We processed the quantification using the National Institutes of Health ImageJ software to analyze the fluorescence intensity.

### Flow cytometry and apoptosis analysis

SC-islets were dissociated into single cells with 0.25% trypsin and washed with precooled PBS. Then cells were subjected to be labeled Annexin V using the Annexin V-647/PI apoptosis detection kit (YEASEN). The death of SC-islet β cells was measured by flow cytometry with Annexin V^+^/INS-GFP^+^ double labeling.

### Insulin secretion assays

The protocol was adapted from a previous study [31]. For GSIS in vitro, SC-islets needed to be fasted in Krebs-Ringer buffer [33] supplemented with 2 mM glucose for 2 h in a 37 °C, 5% CO_2_ incubator. Then the SC-islets were stimulated alternately by Krebs-Ringer buffer with low (2 mM) or high (20 mM) glucose. Supernatants were collected after 30 min of each stimulation and the pellets were lysed overnight in acidified alcohol (75% alcohol, 1.5% HCl) at −20 °C, for insulin content measurement. Secreted insulin or total insulin content was measured by a human insulin ELISA kit (ALPCO).

### Western blot

The protocol was performed as described previously [31], using primary antibodies and secondary antibodies. Primary antibodies were listed as below: β-actin (Beyotime, AF5001, 1:5000), PERK (CellSignalingTechnology, 3192S, 1:1000), eIF2α (Beyotime, AF6771, 1:1000), P-eIF2α (Beyotime, AF1237, 1:1000), ATF4 (Proteintech, 10835-1-AP, 1:500). Secondary antibodies were available from Cell Signaling Technology.

### ISRIB administration

ISRIB solution was made by dissolving 5 mg ISRIB in 2 ml dimethylsulfoxide (DMSO). The solution was kept at RT throughout the experiment. The vehicle solution consisted of DPBS containing of 10% DMSO. ISRIB was given at 100 nM as final concentration in cell culture medium. And ISRIB was given at 2.5 mg/kg/day through i.p. injections for mice from 3 weeks old for 5 weeks. Animals were randomized using randomized number table to be treated with DMSO or ISRIB. All animals received food and water ad libitum.

### Total protein synthesis assay

Cells were plated and recovered overnight, and cell medium was replaced with OPP working solution (1:1000, 20 μM in cell culture medium). After 30 min, cells were washed once with DPBS and fixed with 4% PFA for 30 min at room temperature (RT), and then stained with Click-iT™ Plus OPP Alexa Fluor™ 647 protein synthesis kit (Invitrogen, C10458). Additional immunostaining could be processed following OPP staining.

### Animal studies

All experiments were performed in accordance with the University of Health Guide for the Care and Use of Laboratory Animals and approved by the Biological Research Ethics Committee of Tongji University. The in vivo experiments were blinded to data collectors.

### Generation of conditional Wfs1 knockout mice

Mice were obtained as described [21, 37]. Briefly, *Wfs1*^*fl/fl*^ mice were generated by Shanghai Model Organisms Center, Inc (Shanghai, China) using the CRISPR-Cas9 technology. *Pdx1-Cre* mice were ordered from Shanghai Model Organisms Center, Inc (Shanghai, China). All mice were maintained in the animal facility of Tongji University, Shanghai, China. Mice were maintained on the C57BL6 background, housed in standard cages, fed a normal diet and maintained in a 12-h light/dark cycle. PCR primers were designed to verify the correctly targeted allele (Supplementary Table 1).

### Intraperitoneal glucose tolerance (IPGTT) Test

Mice were fasted for 16 h before the experiment. 20% (w/v) Glucose solution were administered intraperitoneally at a dose of 2 g/kg body weight. Blood glucose levels were measured at 0, 15, 30, 60, and 120 min, respectively, from the tail vein.

### Glucose-stimulated insulin secretion (GSIS) in vivo

Mice were fasted for 16 h before the experiment. 20% (w/v) Glucose solution were administered intraperitoneally at a dose of 2 g/kg body weight. Blood samples were collected from the venous sinus immediately before glucose administration and after 20 min to measure blood insulin levels. Blood was put on ice for 1 h, and centrifuged for 15 min to collect serum. Serum insulin levels were measured using a mouse insulin ELISA kit (ALPCO) according to the manufacturer’s instructions.

### Statistical analyses

All statistical analyses were performed using GraphPad Prism® 8.0/9.0 (GraphPad Software, San Diego, CA). Western blots were quantified by densitometric analyses using ImageJ software and standardized to β-actin. Sample sizes were chosen based on availability of experimental samples. The sample sizes were sufficient since we used many experimental techniques to confirm the results. Statistical methods were not used to determine sample size on animal studies. Results represent mean ± SD. All experiments were repeated at least three independent biological replicates. Comparisons between groups were calculated using unpaired, two-tailed, Student’s *t* test (two groups) and one-way ANOVA or two-way ANOVA (multiple groups). *p < 0.05, **p < 0.01, ***p < 0.001, n.s. not significant.

### Supplementary information

Supplementary Information

Figure S1

Figure S2

Figure S3

Figure S4

Figure S5

Figure S6

Figure S7

Supplementary Table 3

Supplementary Table 4

Supplementary Table 5

## Data Availability

The raw and processed data used for this study have been deposited on GEO with accession numbers GSE235331.

## Code availability

Code for the analysis used in this study have been deposited on GitHub at https://github.com/xychen24/Code_for_ISRIB
