# Supplementary material for: ISR inhibition reverses pancreatic β-cell failure in Wolfram syndrome models
Source: Cell Death Differ. 2024 Feb 6;31(3):322–34. doi: 10.1038/s41418-024-01258-w (PMC10923889; doi:10.1038/s41418-024-01258-w)
Supplement: Supplementary file 1 — Supplementary Information [file 41418_2024_1258_MOESM1_ESM.docx]

**Supplemental Fig.1 Generation and characterization of *WFS1*-deficient hESCs.**

**A and B** Schematic representation of *WFS1* showing the position and deletion region of *WFS1^-/-^* hESCs. Mutation site is highlighted in blue color. **C** Representative bright field images and cell proliferation of WT and *WFS1^-/-^* hESCs. Scale bar, 100 μm. **D** H&E staining of teratoma tissue derived from WT and *WFS1^-/-^* hESCs. Scale bar, 100 μm. Data are presented as the mean ± SD.

**Supplemental Fig.2 Quality control of scRNA-seq datasets of SC-islet between WT and *WFS1^-/-^*.**

**A and B** Violin plots showing the distribution of gene numbers, total counts and percentage of mitochondrial genes for individual cells in WT SC-islet before and after quality control. **C and D** Violin plots showing the distribution of gene numbers, total counts and percentage of mitochondrial genes for individual cells in *WFS1^-/-^* SC-islet before and after quality control. The criteria for quality control is cells with less than 200 or more than 9000 detected genes, or fraction of reads overlapping mitochondria gene more than 10% were excluded from further analysis.

**Supplemental Fig.3 Cell type identification of captured single cells.**

**A** UMAP plot of 6051 retained single cells. Each dot represents a single cell, and cells are colored by original clusters. UMAP_1 and UMAP_2 are outputs of Uniform Manifold Approximation and Projection to capture and visualize the relationship in a lower dimensional space. **B** The expression level of key marker genes in each cell type. **C** Feature plots showing expression level of marker genes for each cell type, β cells (*INS, PCSK1 and G6PC2*), α cells (*GCG*), δ cells (*SST*), ε cells (*GHRL*), pancreatic progenitor cells (*SOX9*), proliferation cells (*MKI67*), EC cells (*FEV*).

**Supplemental Fig.4 Reactome pathway enrichment analysis of genes differentially expressed between WT and *WFS1^-/-^*** **β cells.**

**A** Functional enrichment analysis of up-regulated genes in β cells. The bar plot shows the top 25 significantly enriched pathways. The pathways that highly associated with cell response to stress are highlighted in red. **B** Functional enrichment analysis of down-regulated genes in β cells. The bar plot shows the top 25 significantly enriched pathways. The pathways that highly associated with translation are highlighted in red.

**Supplemental Fig.5 The heatmap of UPR associated genes between WT and** ***WFS1^-/-^* β cells.**

**A** Average expression of signature genes in UPR genes between WT and *WFS1^-/-^* β cells.

**Supplemental Fig. 6 Signature protein pattern of PERK/eIF2α pathway was determined by western blot analysis in WT and *WFS1*^-/-^ SC-islets.**

**A** Origin western blots.

**Supplemental Figure 7. The immunostaining of Wfs1 and Ins showing the** **efficient knockout of Wfs1 in CKO mice.**

**A** and **B** Immunostaining of Wfs1 and Ins (**A**) and quantifications (**B**) of Wfs1^+^Ins^+^ cells in WT and CKO mice after ISRIB treatment, n = 9 islets from n = 3 mice. Scale bar, 25 μm. **C** H&E staining of organs in WT and CKO mice after ISRIB treatment. Scale bar, 100 μm. Data are presented as the mean ± SD. *p* values calculated by unpaired two-tailed Student’s *t* test were **p* < 0.05, ***p* < 0.01 and ****p* < 0.001.

**Supplemental Tables.**

**Supplemental Table 1. Primer used for qPCR and genotyping**

| Primer | Sequence (5’-3’) |
| --- | --- |
| Q-*WFS1*-F | GGAAGCTCAACCCCAAGAAG |
| Q-*WFS1*-R | CCAGCTCGTCATCATCTTCG |
| *Wfs1^fl/fl^*-F-1 | TGCCCTCCAAAGCCACACTG |
| *Wfs1^fl/fl^*-R-1 | GGCCCTGCTACCTACTCCCATTTT |
| *Wfs1^fl/fl^*-F-2 | GTCCGACACACATCCTGTCT |
| *Wfs1^fl/fl^*-R-2 | TTCCCGGTGTACTTGGGTCT |
| *Pdx1*-*Cre*-F | CTGGACTACATCTTGAGTTGC |
| *Pdx1*-*Cre*-R | GGTGTACGGTCAGTAAATTTG |

**Supplemental Table 2. Top 10 markers among different cell types in WT and *WFS1^-/-^* SC-islet.**

| β cells | α cells | ε cells | δ cells |
| --- | --- | --- | --- |
| RBP4 | GCG | GHRL | HHEX |
| INS | GC | GFRA3 | CRH |
| HADH | TMSB4X | CELA3A | ST3GAL1 |
| PCSK1 | SERPINI1 | AL590556.3 | CBLN2 |
| INS-IGF2 | TTR | ACSL1 | SST |
| IGF2 | NRCAM | SPINK1 | LEPR |
| AC132217.2 | NKAIN4 | DEPP1 | SAMD5 |
| AC132217.1 | VIM | SDC2 | PCP4 |
| PLAGL1 | GSTA1 | CRH | ERO1B |
| FABP1 | LBH | ITM2A | ISL1 |

| Pancreatic progenitor | Polyhormonal cells | Proliferation cells | EC cells |
| --- | --- | --- | --- |
| CCND1 | NBL1 | TOP2A | CBLN1 |
| ANXA2 | AP001453.3 | CENPF | FEV |
| GP2 | FKBP2 | MKI67 | COL5A2 |
| SLC4A4 | AC020907.6 | HMGB2 | PALM2-AKAP2 |
| CFTR | FXYD3 | NES | SYT13 |
| ENC1 | C4orf48 | SMC4 | DDC |
| CALB1 | CTSZ | TUBA1B | CALB2 |
| KRT18 | DPP7 | TUBB | CHGA |
| DLK1 | NDUFA13 | HIST1H4C | CRYBA2 |
| SPINK1 | CTSD | VIM | GPC3 |

**Supplemental Table 3. Genes that are significantly branch dependent.**

**Supplemental Table 4. Differential expression genes between *WFS1^-/-^* and WT.**

**Supplemental Table 5. Reactome pathway enriched by differential expression genes between *WFS1^-/-^* and WT.**
