# Supplementary figures and images for: ISR inhibition reverses pancreatic β-cell failure in Wolfram syndrome models
Source: Cell Death Differ. 2024 Feb 6;31(3):322–34. doi: 10.1038/s41418-024-01258-w (PMC10923889; doi:10.1038/s41418-024-01258-w)

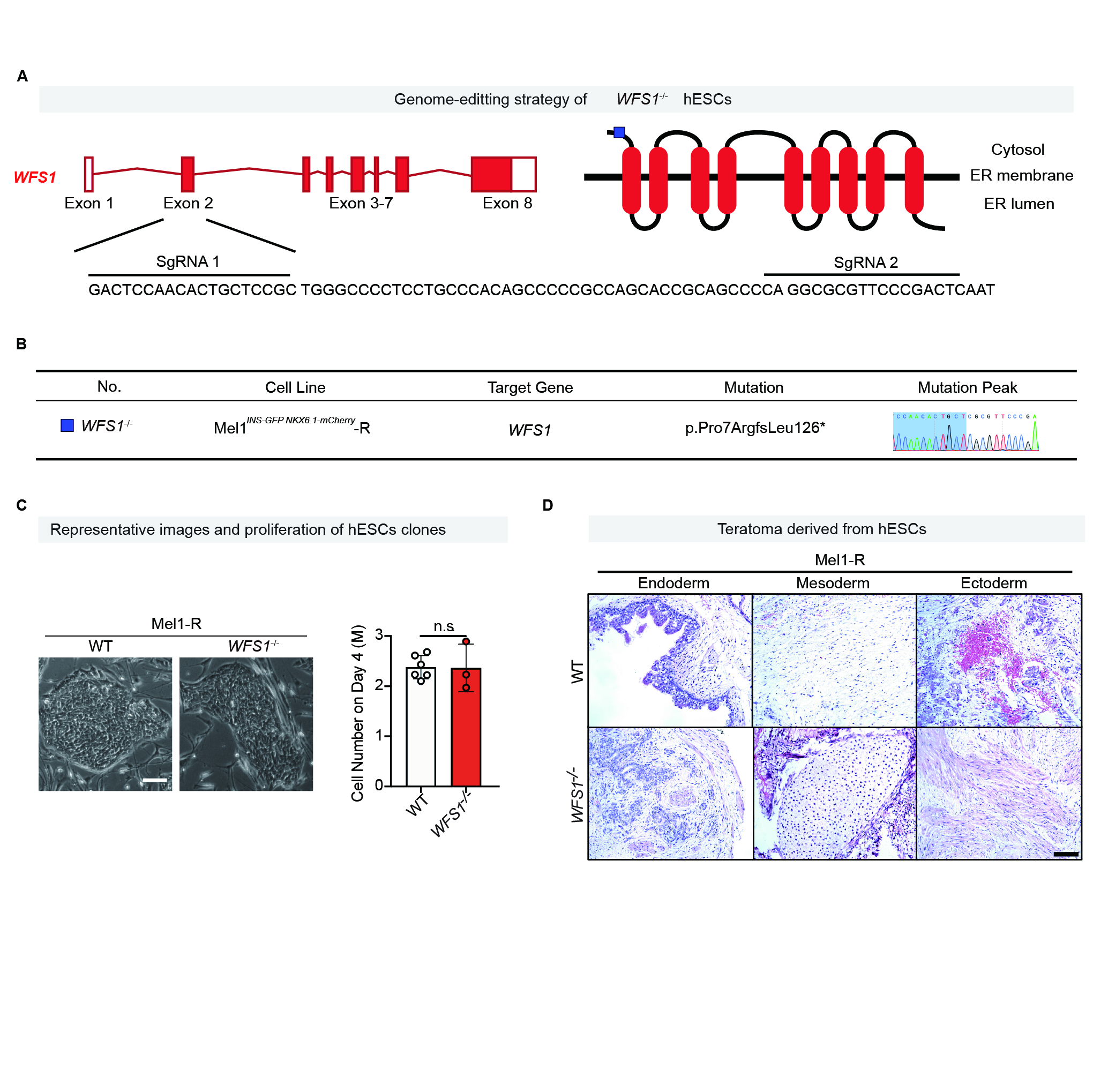

Supplement: Supplementary file 2 — Figure S1 [file 41418_2024_1258_MOESM2_ESM.tif]

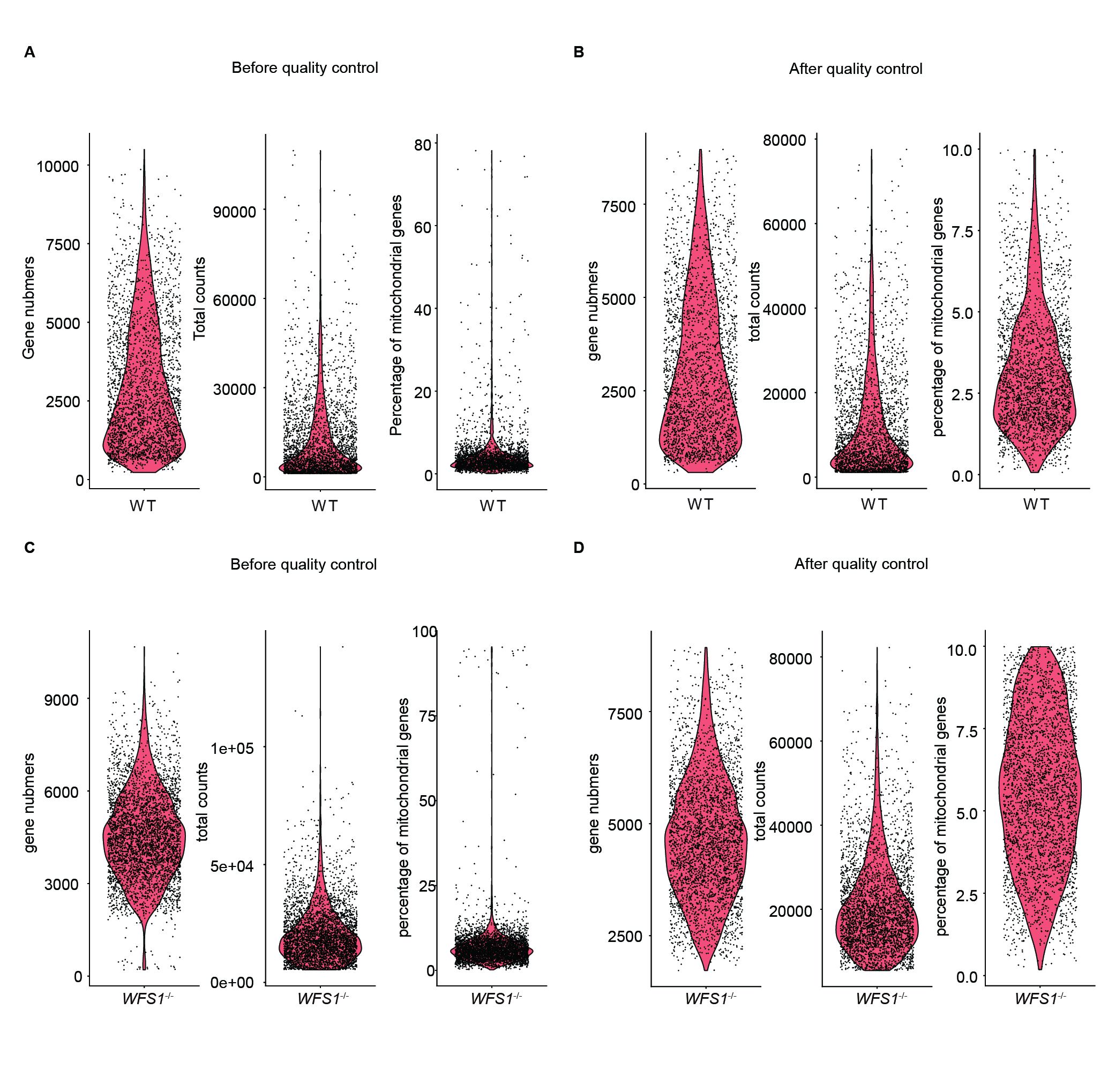

Supplement: Supplementary file 3 — Figure S2 [file 41418_2024_1258_MOESM3_ESM.tif]

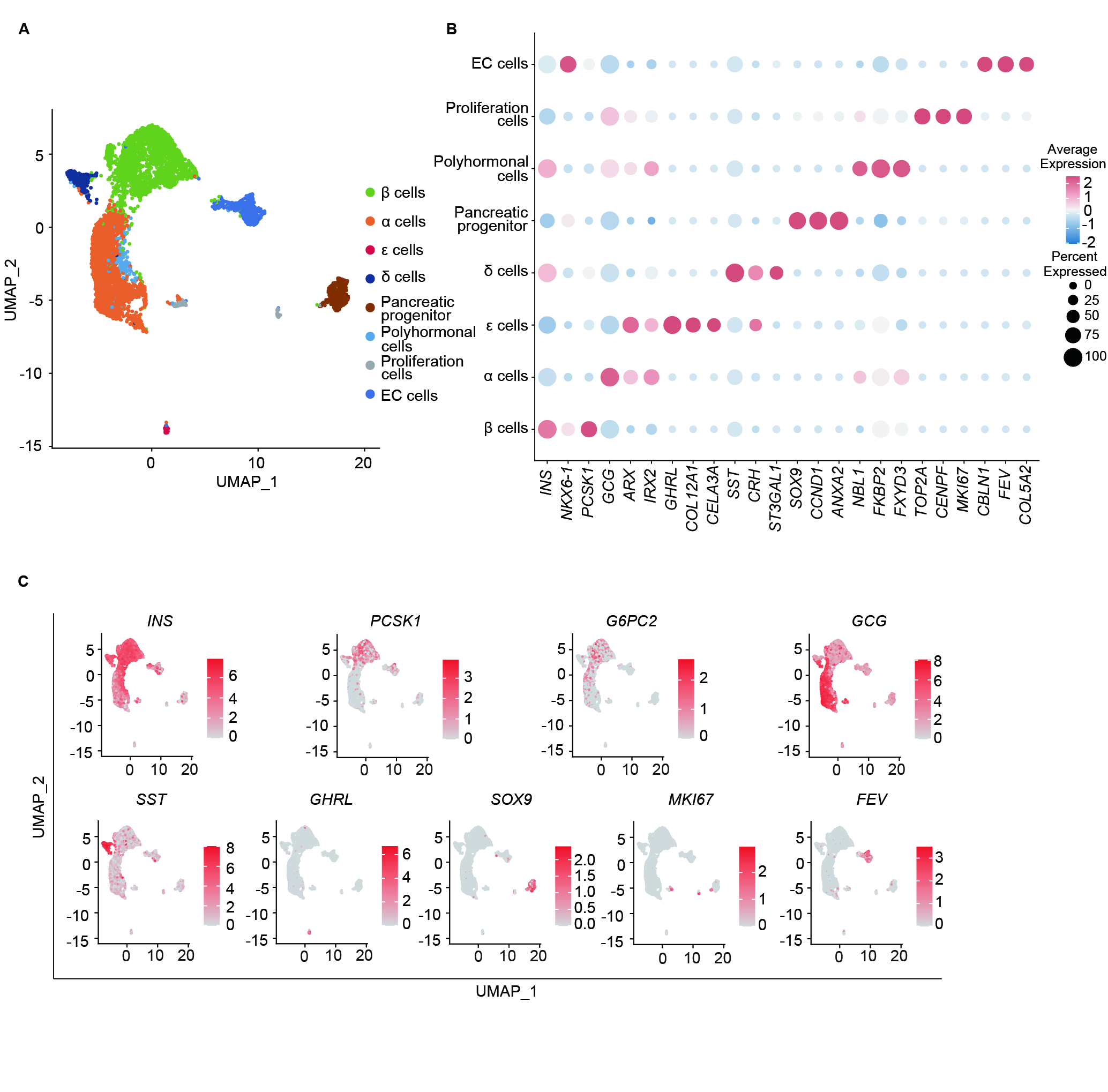

Supplement: Supplementary file 4 — Figure S3 [file 41418_2024_1258_MOESM4_ESM.tif]

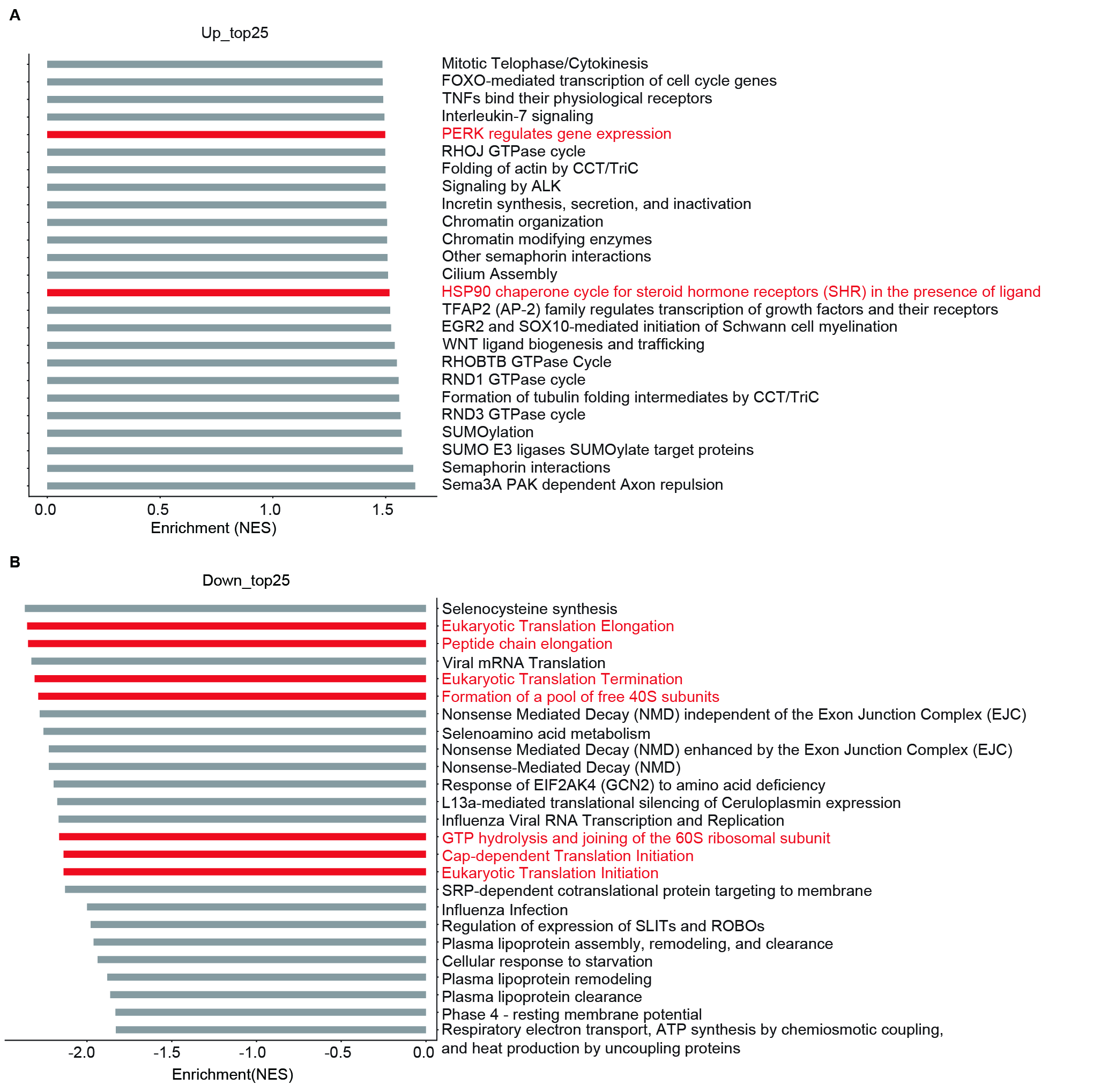

Supplement: Supplementary file 5 — Figure S4 [file 41418_2024_1258_MOESM5_ESM.tif]

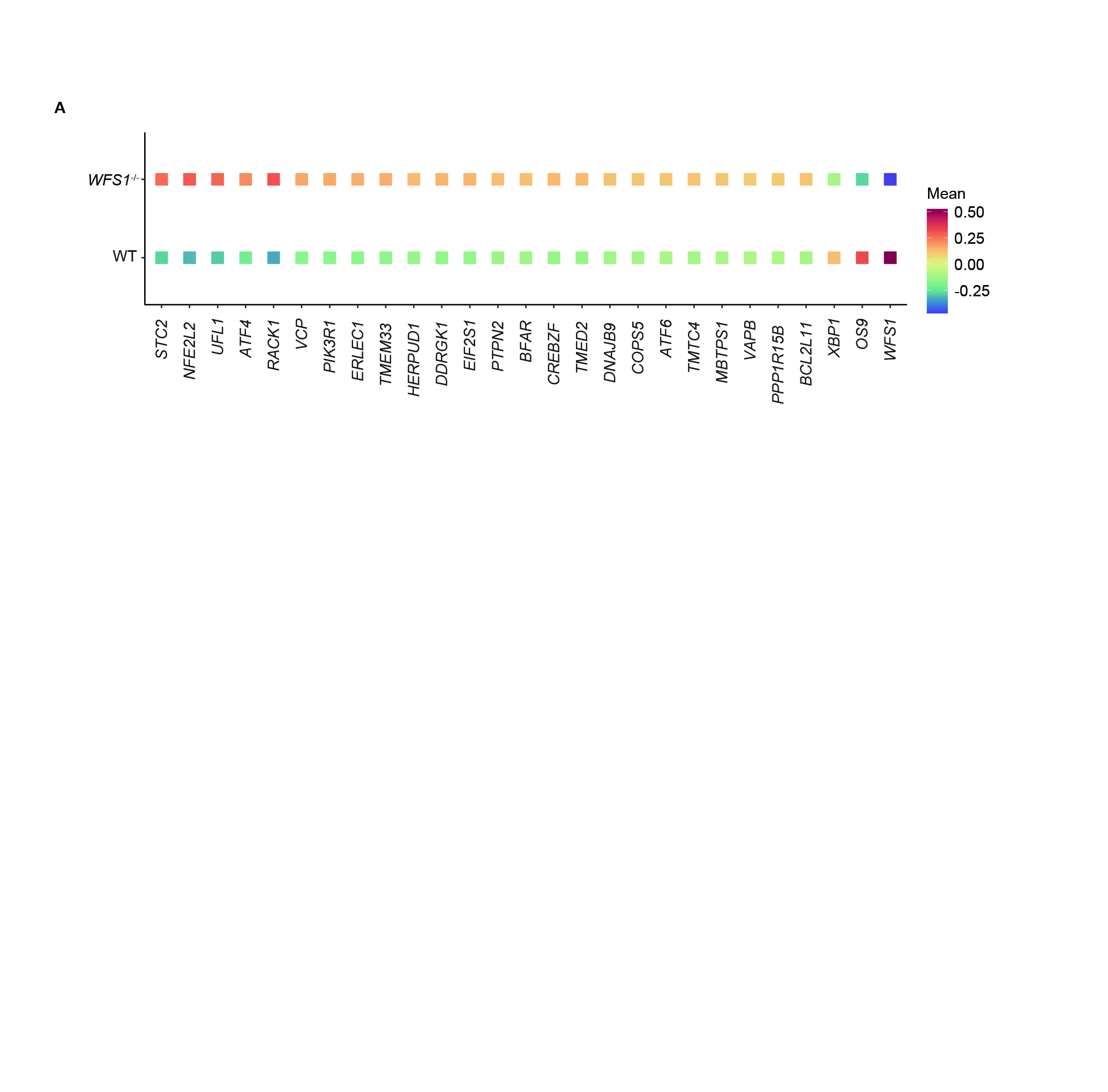

Supplement: Supplementary file 6 — Figure S5 [file 41418_2024_1258_MOESM6_ESM.tif]

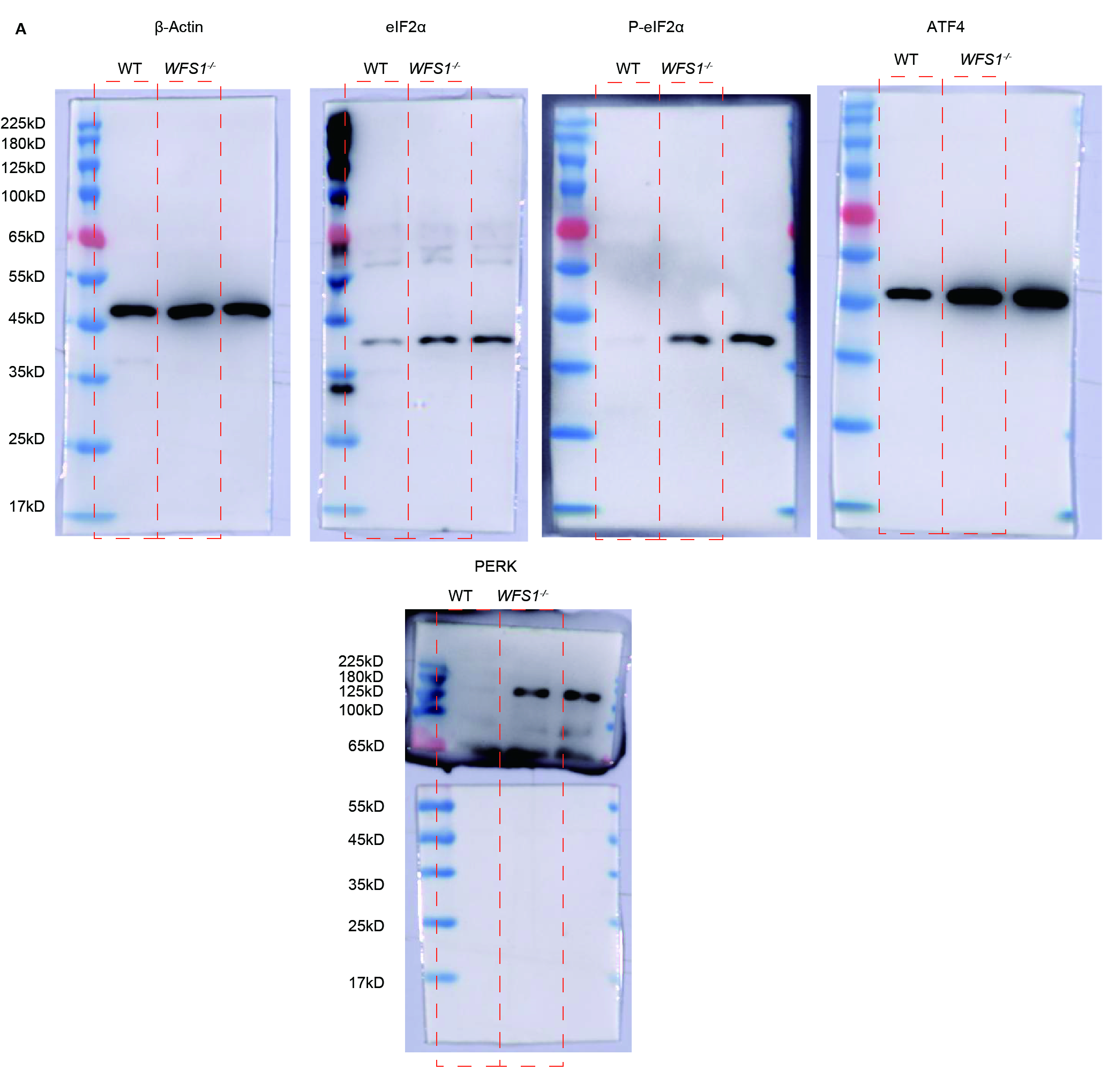

Supplement: Supplementary file 7 — Figure S6 [file 41418_2024_1258_MOESM7_ESM.tif]

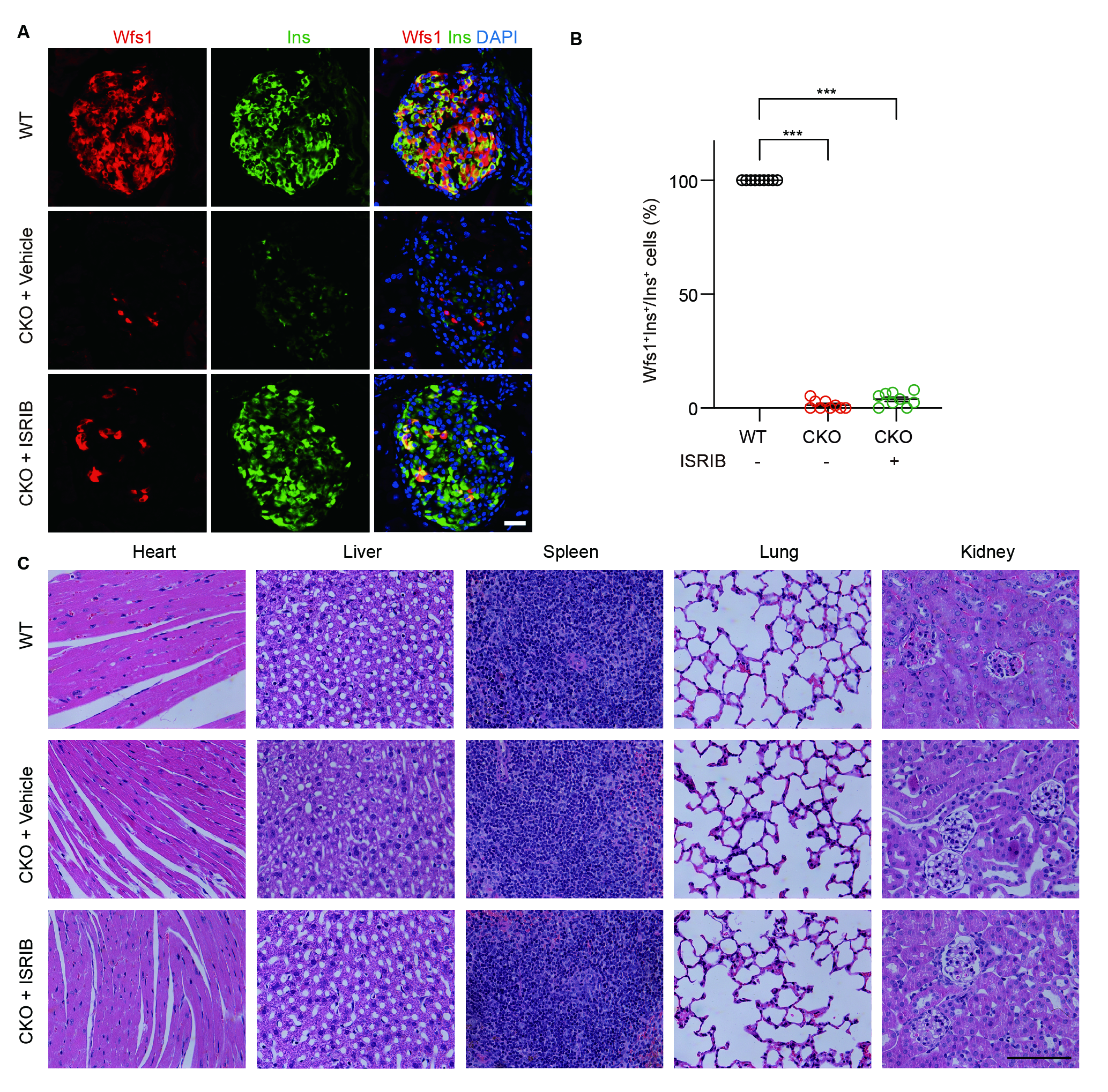

Supplement: Supplementary file 8 — Figure S7 [file 41418_2024_1258_MOESM8_ESM.tif]
